# Text Messaging Interventions for Improvement in Physical Activity and Sedentary Behavior in Youth: Systematic Review

**DOI:** 10.2196/10799

**Published:** 2018-09-17

**Authors:** Kim Ludwig, Rosie Arthur, Nicholas Sculthorpe, Hollie Fountain, Duncan S Buchan

**Affiliations:** 1 Institute of Clinical Exercise and Health Science School of Health and Life Sciences University of the West of Scotland Blantyre United Kingdom; 2 School of Applied Sciences Edinburgh Napier University Edinburgh United Kingdom

**Keywords:** review, exercise, sedentary lifestyle, text messaging, cell phone, telemedicine, adolescent

## Abstract

**Background:**

The use of text messages (short message service, SMS) to change physical activity and sedentary behavior in youth is of interest due to the need for novel, more effective intervention approaches. Previous reviews have examined a variety of technology-based interventions and their impact on different health behaviors, but evidence regarding the impact of just SMS on physical activity and sedentary behavior is lacking.

**Objective:**

The aim of this study was to assess the effectiveness and use of theory of SMS interventions for improving physical activity and sedentary behavior in youth.

**Methods:**

Authors systematically searched electronic databases from March to November 2017. Citations were sifted using additional reviewers, and a qualitative synthesis of eligible studies was conducted using piloted data extraction forms. To be eligible for inclusion, studies had to be of a randomized controlled or quasi-experimental design, incorporate SMS, involve adolescents between the ages of 10 and 19 years, and assess at least one physical activity or sedentary behavior outcome. Risk of bias was assessed using the Cochrane Collaboration’s Risk of Bias tool.

**Results:**

A total of 13 studies reporting 11 interventions were included in the qualitative analysis. Studies included interventions that were conducted in schools, online, or face-to-face. Studies were of high heterogeneity with regard to study duration, participant characteristics, intervention content, and outcome measures. Findings were equivocal with regard to intervention effectiveness for physical activity and sedentary behavior. Overall, 7 interventions resulted in an improvement for physical activity and 6 for sedentary behavior. All studies were judged to be of high risk of bias for at least 1 item.

**Conclusions:**

Some studies in this review showed promising results for using SMS to improve physical activity and sedentary behavior in youth. High heterogeneity of design and outcome measures precluded data pooling and conclusions as to which specific intervention elements are linked to increased effectiveness cannot be drawn. The authors propose incorporating the following elements in future studies: specific focus on desired health behavior; mixed-methods design; include long-term follow-up; include self-monitoring, goal setting, and feedback; combine SMS with a mobile app; and send 3 or more SMS text messages per week. More rigorous studies are needed to explore the relationship between intervention effectiveness and specific intervention components such as content and delivery.

## Introduction

### Physical Activity and Sedentary Behavior

Participating in sufficient levels of physical activity (PA) is essential to reduce the risk of all-cause mortality and cardiovascular disease [1,2]. For adolescents, it is recommended that they undertake at least 60 min of moderate to vigorous PA (MVPA) per day [3]. Unfortunately, few adhere to these current activity recommendations with adolescence characterized by declining PA levels in conjunction with increased sedentary time, despite calls for sedentary time to be minimized [4]. For instance, findings from Europe suggest that 83.2% of the adolescents aged 11 to 17 years do not achieve a minimum of 60 min of MVPA per day, whereas globally, it has been estimated that 80.3% of adolescents are insufficiently active [5]. Moreover, global data suggest that adolescents spend 57% of their time in sedentary activities, with 40% of adolescents spending 3 or more hours watching television on weekdays, increasing up to 50% on weekends [6,7]. These findings are particularly concerning as sedentary behavior (SB) is associated with various aspects of poor psychological and physiological health and all-cause and cardiovascular disease-related mortality [8-11]. Conversely, increased PA improves adiposity, blood lipid profile, blood pressure, insulin resistance, aerobic fitness, and bone health [12] while also reducing premature all-cause mortality [13]. Given these relationships, both SB and PA are important therapeutic targets to reduce lifestyle-induced noncommunicable diseases and especially during adolescence, as behaviors developed in younger ages are likely to continue into later life [14,15]. Given the inconsistent success of traditional intervention approaches, there is a need for research to generate new strategies to modify physical inactivity and SB [16].

### Mobile Health

Mobile health (mHealth) which draws upon mobile devices for health-related apps has emerged as a promising tool for health-related behavioral interventions [17]. Mobile phones are used by all age groups, with more than 90% of UK children aged 12 to 15 years currently using them [18]. Such high usage suggests that these mobile devices may offer a cost-effective and acceptable means for delivering health behavior change interventions that can fit within people’s everyday lives and have population-wide reach. Unsurprisingly, mHealth approaches are also being used to provide health care services worldwide, including Africa, Asia, and South America [19]. In the United Kingdom, the National Health Service is employing the SMS (short message service) text messaging system Florence to support patients in monitoring, managing, and improving their health [20]. mHealth systems can also be used to send appointment or medication reminders to support health care workers by providing training, decision making, and communication tools as well as to implement health promotion and educational interventions [19,21]. However, there is a lack of evidence regarding the effectiveness of mHealth interventions on behavior changes and health outcomes [19,22,23]. Unfortunately, research that has examined the effects of SMS interventions on PA and SB in youth is also scant.

Previous systematic reviews and meta-analyses involving adolescents have included a variety of technologies, such as apps, email, video games, and websites when reviewing the evidence on the most effective means of improving PA and SB [24-32]. However, none of these reviews have assessed the effectiveness of SMS in isolation. Moreover, reviews have included a number of outcomes such as disease state or medication adherence [25,33-36] and have focused on several different health behaviors, such as smoking and diet [25,27,29-32,34]. As such, evidence that has examined the efficacy of mobile devices to influence PA and SB is lacking. Furthermore, and to the best of our knowledge, existing systematic reviews and meta-analyses involving adolescents and SMS as a means for improving PA and SB have not explored the use of theoretical frameworks [24,30-32,34-37].

### Theoretical Frameworks

As evidence has shown the increased effectiveness of health interventions using a behavioral theory framework [38,39], it is surprising that many interventions have been developed without a proper underpinning theory. Even in those studies that suggest their intervention was informed by appropriate theory, the specific application of theory often remains unclear [40,41]. In addition to evaluating the evidence of the effectiveness of interventions using mobile phones for improving PA and SB, it is important to evaluate the theory and behavior change techniques (BCTs) that have been used to develop these interventions. Providing this information is essential for health care practitioners to ensure that future mHealth interventions are effectively implemented.

### Aims

To provide this evidence, this review aimed to systematically identify mHealth studies that have been developed to increase PA levels and to reduce SB in adolescents. A subsequent aim was to identify the theory and BCTs used in these studies. Findings from this review are expected to provide an insight into the development of future mHealth interventions to maximize their effectiveness.

## Methods

### Data Reporting

All data are reported in accordance with the Preferred Reporting Items for Systematic Reviews and Meta-Analyses statement guidelines [42].

### Eligibility Criteria

Experimental (randomized controlled trial or quasi-experimental design) studies were included if they involved or reported data separately for participants between the ages of 10 and 19 years with or without known morbidities; used SMS via a mobile phone within the intervention, both in addition to other intervention components or on its own; employed usual care, another intervention, or no intervention as comparator; and assessed at least one outcome related to PA or SB. All outcomes related to PA and SB, such as step count, moderate PA (MPA), and screen time, as well as all subjective and objective outcome measures were eligible for inclusion.

Furthermore, only studies that were written in the English language and where full text was available were included. Studies were excluded if they solely used other technologies such as apps, websites, or email.

### Information Sources

A systematic search of the following electronic databases was conducted in March 2017 and updated in November 2017: Web of Science (coverage 1864-2017), PubMed (1809-2017), MEDLINE (1946-2017), Cumulative Index to Nursing and Allied Health Literature Complete (1937-2017), PsycINFO (1800s-2017; not available for search update and replaced by PsycARTICLES 1894-2017), and SPORTDiscus (1930-2017). All databases except PubMed (November 7, 2017) were last searched on November 8, 2017. During the initial search, KL searched bibliographies and contacted corresponding authors of eligible studies. Bibliographies of existing systematic reviews and meta-analyses identified during the initial search process were also screened for eligible studies [24-37,43,44].

### Search

Search terms and combinations of the electronic database search are shown in Table 1.

### Study Selection

Study citations from the electronic search were imported into the reference manager software Zotero (Version 5.0, online and standalone). KL manually removed duplicates. For the initial search, KL and HF independently screened titles and abstracts of all remaining studies. Following the search update, KL and DSB independently reviewed new titles and abstracts with the full texts of relevant titles obtained to confirm eligibility. KL and HF (DSB for search update) discussed discrepancies until consensus was reached. KL hand-searched bibliographies of eligible studies and contacted corresponding authors for additional manuscripts. All eligible studies were then included in the qualitative analysis.

### Data Collection Process

Data extraction was conducted based on the Cochrane Collaboration’s Data Extraction Template for Included Studies (Version 1.8) [45]. Items of interest for this review such as the content of SMS and interactivity were added to the Cochrane Data Extraction Template. KL piloted the updated template on 2 randomly chosen studies eligible for this review. Subsequently, the piloted form was revised where necessary. Thereafter, KL and HF (DSB after search update) independently extracted required data using the revised form. Extractions were compared and discussed until consensus was reached for all items. Content was then synthesized for analysis.

### Data Items

Data extracted included (1) general study information (such as country, aims, and target health behavior); (2) methods (such as study design and duration of intervention); (3) participants (such as population description, number recruited, age, sex, and health status); (4) intervention and control groups (such as name of group, number of participants randomized, intervention mode, content, use of theory, message content, frequency, device, interaction, and adherence); (5) outcomes (assessed PA and SB outcomes, method of PA/SB outcome assessment, timing of PA/SB outcome assessment); (6) results and conclusion (including additional results information and relevant conclusions); (7) other information (including funding source and conflicts of interest). Where data were missing or clarification was sought, study authors were contacted. Where multiple studies reported on multiple follow-up periods or outcomes of the same intervention, outcomes from the longest follow-up time point available for each outcome were extracted.

### Risk of Bias in Individual Studies

Assessment of risk of bias was conducted at study level. KL and HF (DSB after search update) reviewed all included manuscripts using the Cochrane Collaboration’s risk of bias assessment tool [46]. KL employed this assessment tool using RevMan (software, version 5.3). Due to the nature of behavioral interventions, blinding of participants and personnel is challenging and rarely incorporated [47]. This item was therefore not included in the assessment. The following remaining domains were judged: selection bias (random sequence allocation and allocation concealment), detection bias (blinding of outcome assessment), attrition bias (incomplete outcome data), reporting bias (selective reporting), and other bias. KL and HF (DSB after search update) ranked each item as high, low, or unclear risk for each study and discussed discrepancies until a consensus was reached.

**Table 1:** Electronic database search terms and combinations. Asterisks were used to search for words beginning with these letters.

Category		Search term
**Intervention mode**		
	1	“mobile phone”
	2	smartphone
	3	“cell phone”
	4	“handheld device”
	5	text messag*
	6	SMS^a^
	7	“messag* service”
	8	“messaging system”
	9	mHealth
	10	telehealth
	11	“online health”
	12	e-Health
	13	eHealth
	14	“mobile health”
	15	“digital media”
	16	ICT^b^
	17	(1-16) combined with OR
**Study design**		
	18	“randomised controlled”
	19	“randomized controlled”
	20	RCT^d^
	21	“controlled trial”
	22	quasi-experimental
	23	(18-22) combined with OR
**Participants**		
	24	adolescen*
	25	youth
	26	“young people”
	27	“young adult*”
	28	child*
	29	paediatric
	30	pediatric
	31	teen*
	32	“school age”
	33	“school-aged”
	34	highschool
	35	“secondary school”
	36	(24-35) combined with OR
**Behavior**		
	37	activity
	38	sport
	39	exercise
	40	health*
	41	“behaviour change”
	42	lifestyle
	43	sedentary
	44	sitting
	45	(37-44) combined with OR
	46	(17,23,36,45) combined with AND

^a^SMS: short message service.

^b^ICT: information and communication technology.

^c^RCT: randomized controlled trial.

## Results

### Study Selection

**Figure 1:**
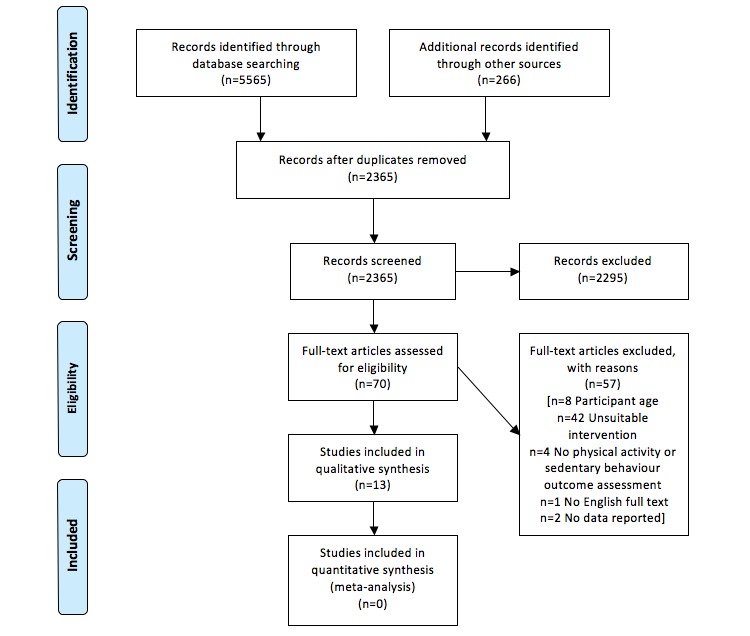
Literature search flow chart.

The electronic database and hand search produced 5565 and 266 studies, respectively. After removal of duplicates, 2365 studies were screened. A total of 2295 records were excluded, and 70 full-text articles were assessed. Moreover, 13 eligible full-text articles assessing 11 different interventions remained and were included in the qualitative analysis. A flowchart of the systematic literature search is displayed in Figure 1.

### Study Characteristics

Study characteristics of included studies are shown in Tables 2 and 3. A total of 12 studies targeted PA [48-59] and 7 targeted SB [48-51,54,59,60]. Additionally, most studies also focused on dietary behaviors [49-52,54,57,59,60].

Some studies focused on participants with specific characteristics, including those not meeting current PA guidelines [48,53], not participating in physical education lessons or organized sports [54], having type 1 diabetes [56], being at high risk for diabetes [57], having a body mass index ≥ the eighty-fifth percentile [49,59], and being ≥1 year post cancer therapy [55]. When including overweight or obese participants, rates ranged between 23.7% (62/262) [52] and 55% (22/40) [49] for overweight and between 6.7% (15/225) [52] and 45% (18/40) [49] for obesity. The mean age of participants ranged between 12.5 [52] and 17.3 years [58]. One intervention only included female participants [50,51,54]. A total of 12 studies consisted of ≥50% female participants [48,50-60].

### Intervention Design and Content

A total of 2 interventions included SMS in addition to a school program [50-52,54]. A total of 5 interventions used SMS text messages as part of an online intervention [49,53,55,57,60] and others used pedometers [56], group sessions and telephone calls [59], apps [48,49,55], and Fitbit trackers (Fitbit, Inc.) [49,55]. Only one intervention consisted solely of SMS [58]. Moreover, 2 interventions consisted of different types of SMS [48,58]. Depending on group allocation, one employed SMS focusing on affective or instrumental beliefs [58], whereas the other involved SMS from different senders, including a parent, peer, or behavioral health specialist [48]. School-based interventions using SMS included elements such as sports and PA opportunities, educational (group) seminars, provision of healthy foods, self-monitoring tools, and printed or email materials promoting healthy lifestyles [50-52,54]. One intervention also used a Facebook group to promote healthy lifestyles and keep participants informed about the intervention [52]. Interventions that included an online component also consisted of a variety of elements, such as forums, diet analysis, videos, educational games, challenges, educational materials, expert advice, behavioral skill training, goal setting, monitoring, feedback, and tutorials on behavioral change strategies [49,53,57,60]. One study included access to a private Facebook group, which provided rewards for achievements, encouragement, and a discussion board, as well as using Fitbit trackers and an app to monitor progress toward individualized goals [55].

In another study, participants wore pedometers that were used to encourage PA and facilitate recording progress [56]. Another study included group sessions that provided education on health behaviors and achieving successful behavior change. In this study, participants also received phone coaching during the 12-month maintenance period post intervention [59]. One study using an app for monitoring and reporting of PA also included autonomous and external goal setting as well as daily feedback [48]. Depending on which condition participants were assigned for that day, SMS text messages were sent by a behavioral health specialist, parents, or a peer [48].

### Content of Text Messages

SMS text messages were used to encourage, motivate, reinforce, and prompt participants to be physically active or maintain their current positive behavior changes [48-51,53-56,59,60]. Some studies provided participants with suggestions for healthy lifestyle behaviors [48,49,59].

In addition to promoting PA, one study also employed SMS to provide participants with health behavior information, behavioral skills, and solutions for PA barriers to reinforce the benefits of PA and to build rapport with a virtual friend [53]. SMS text messages were also used for feedback [48,53], which in one study depended on the participant’s goal attainment [48]. SMS also included statements from testimonials as well as messages targeting intrinsic motivation and reflective questioning [59]. SMS text messages were also used to reduce risk behaviors [60]. Two interventions employed SMS aiming to increase participant self-efficacy [59,60]. Three interventions sent SMS related to goal-setting, such as the participants’ specific weekly challenges [55,57,59]. In addition to this, one intervention included affective SMS for encouragement and as a reminder of PA goals. In this intervention, SMS text messages sent in intervention week 2 were based on the participants’ step counts from week 1 [55]. Another study sent SMS text messages regarding affective or, depending on the intervention group, instrumental gains associated with regular PA. These include messages regarding the benefits of being active, such as physical and psychological improvements [58]. Three studies used SMS text messages to remind participants to follow the intervention protocol, such as logging on to the intervention website or wearing an activity tracker [49-51,53,54,56,57].

### Theory Derivation

Three studies based their interventions on the transtheoretical model (TTM) of behavior change or stage of motivational readiness for change (SOC) model [53,57,60]. One study used the SOC model to tailor intervention content and presentation, such as by adapting TM and website content according to the participant’s stage of motivational readiness [53]. Participants in precontemplation, contemplation, and preparation stage were given information on benefits and barriers of PA, opportunities for PA, goal setting, as well as PA planning. Participants classed in the action stage were provided with monitoring tools and information to prevent relapse [53]. In addition to the TTM, one study also used the I-Change, Attitude-Social Influence-Self-Efficacy model and addressed attitude, social influence, and self-efficacy. They emphasized the advantages of following the recommendations and disadvantages of risk behaviors, created a healthy online social environment, and strengthened skills to avoid risk behaviors [60].

**Table 2:** Study characteristics of included studies—sample and outcomes.

Author, year, country	N^a^	Design	Age, mean (SD)	PA^b^ and SB^c^ outcomes	Assessment
Brannon et al, 2017, United States [48]	10	N-of-1 RCT^d^	16.7 (0.95)	MVPA^e^ min/day, SB min/day	Objective
Chen et al, 2017, United States [49]	40	RCT	14.9 (1.7)	PA days/week, TV/computer hours/day	Self-report
Dewar et al, 2013, Australia [50]	357	Group RCT	13.2 (0.5)	Accelerometer counts/min, % MVPA, screen time min/day	PA: objective; SB: self-report
Dewar et al, 2014, Australia [51]	357	Group RCT	13.2 (0.5)	% MPA^f^, VPA^g^, MVPA; SB min/day	PA: objective; SB: objective + self-report
Ermetici et al, 2016, Italy [52]	487	Nonrandomized CT^h^	12.5 (0.4)	MVPA hours/week, screen time hours/day	PA: objective + self-report; SB: self-report
Lana et al, 2014, Spain and Mexico [60]	2001	RCT	Pre 13.26 (1.03); Post 12.91 (0.77)	SB (less than 360 min PA/week)	Self-report
Lau et al, 2012, Hong Kong [53]	78	Nonrandomized CT	CG^i^ 13.26 (1.14); IG^j^ 12.29 (0.87)	PA level last 7 days	Self-report
Lubans et al, 2012, Australia [54]	357	Group RCT	13.18 (0.45)	Accelerometer counts/min, MVPA min/day, SB min/day	PA: objective; SB: self-report
Mendoza et al, 2017, United States [55]	60	RCT	16.6 (1.5)	MVPA min/day, SB min/day	Objective
Newton et al, 2009, New Zealand [56]	78	RCT	14.4 (2.37)	Step count, MVPA min/week	Objective + self-report
Patrick et al, 2013, United States [57]	101	RCT	14.3 (1.5)	MVPA min/week, SB hours/day	Self-report
Sirriyeh et al, 2010, United Kingdom [58]	120	RCT	17.3 (0.68)	MVPA metabolic equivalent min/week	Self-report
Straker et al, 2014, Australia [59]	44	Within-subject CT	14.1 (1.6)	SB, light, moderate, vigorous PA min/day	Objective

^a^N: number of participants randomized.

^b^PA: physical activity.

^c^SB: sedentary behavior.

^d^RCT: randomized controlled trial.

^e^MVPA: moderate to vigorous physical activity.

^f^MPA: moderate physical activity.

^g^VPA: vigorous physical activity.

^h^CT: controlled trial.

^i^CG: control group.

^j^IG: intervention group.

Moreover, one study used both behavioral determinants models and TTM to guide intervention design [57]. One study employed affective and instrumental beliefs, as well as the theory of planned behavior (TPB) [58]. Two interventions were informed by social cognitive theory (SCT) [49-51,54]. One focused on self-efficacy, outcome expectation, self-monitoring, skill mastery, and self-regulation capabilities [49]. Another employed SCT by planning social support or change, providing general encouragement and information about the link between behavior and health, and identifying barriers and strategies to overcome these. Specifically, outcome expectations, social support, and self-efficacy were targeted [50,51,54]. Self-determination theory (SDT) formed the basis for 2 interventions [55,59], with one also using goal-setting theory [59]. This intervention focused on the provision of a need-supportive environment to achieve greater self-determination, autonomous motivation, and consequently greater engagement with the desired behaviors. The goal-setting theory was employed to increase autonomous and intrinsic goal setting to predict greater goal attainment and engagement with desired behaviors [59]. The other focused on psychological needs that influence motivation such as competence, autonomy, and relatedness. The Fitbit tracker and app aimed to increase competence and autonomy by providing opportunities to set personalized goals and monitor progress. The Facebook group aimed to enhance relatedness by providing support [55]. Cybernetic control theory (CCT) was used by one study, which included self-regulation strategies defined by goal-setting, self-monitoring, goal review, and feedback [48]. Two studies did not provide any information regarding theory derivation. Authors were contacted and lack of a specific theory base informing SMS was confirmed [52,56].

**Table 3:** Study characteristics of included studies—intervention and comparator.

Author, year	Intervention duration	TM^a^ intervention	Comparators
Brannon et al, 2017 [48]	24 days	TM + mobile app	Mobile app only
Chen et al, 2017 [49]	6 months	TM + Fitbit tracker and app + online program	Online program + pedometer + diary
Dewar et al, 2013 [50]	12 months	TM + school program	Waitlist condensed intervention
Dewar et al, 2014 [51]	12 months	TM + school program	Waitlist condensed intervention
Ermetici et al, 2016 [52]	24 months	TM + school program	No information
Lana et al, 2014 [60]	9 months	TM + online program	Online intervention, limited access online intervention
Lau et al, 2012 [53]	8 weeks	TM + online program	No intervention
Lubans et al, 2012 [54]	12 months	TM + school program	Waitlist condensed intervention
Mendoza et al, 2017 [55]	10 weeks	TM + Fitbit tracker and app + Facebook group	Standard care
Newton et al, 2009 [56]	12 weeks	TM + pedometer	Standard care
Patrick et al, 2013 [57]	12 months	TM + online program	Online program, online program + group sessions + phone calls, usual care
Sirriyeh et al, 2010 [58]	2 weeks	TM only	Neutral TM
Straker et al, 2014 [59]	12 months	TM + group sessions + phone calls	No intervention

^a^TM: text messaging.

### Text Message Delivery and Interactivity

In 3 studies, SMS text messages were sent weekly [55,56,60], 2 sent daily [48,58], another sent only on weekdays [53], and 2 studies sent 3 or more each week [52,57]. Two studies only sent SMS text messages during the maintenance period following the intervention [49,59]. In one, the number of SMS text messages was reduced from 3 to 1 per week and finally to 1 per month [59]. In the other, SMS text messages were sent biweekly during a 3-month maintenance phase [49]. Another intervention increased the frequency of SMS from weekly to twice per week [50,51,54]. Five studies specified the time of SMS delivery [48,50-52,54,58,59]. SMS text messages were sent at 4 pm at the end of the school day to minimize the risk of cross-contamination [58], close to meal times [52], between 7 pm and 8 pm [48] and depending on the SMS content, such as immediately after school when encouraging PA [50,51,54]. Another study sent SMS on weekday evenings at 6 pm and at 12 pm on weekends. Here, participants were able to choose on which days they wished to receive the SMS [59].

Three studies gave participants the possibility to interact with the research team and reply to the SMS [53,57,59]. Responding was optional; however, one study provided a monetary incentive to do so [53]. Another study also allowed interactivity; however, participants would only receive one reply [59].

### Risk of Bias Within Studies

**Figure 2:**
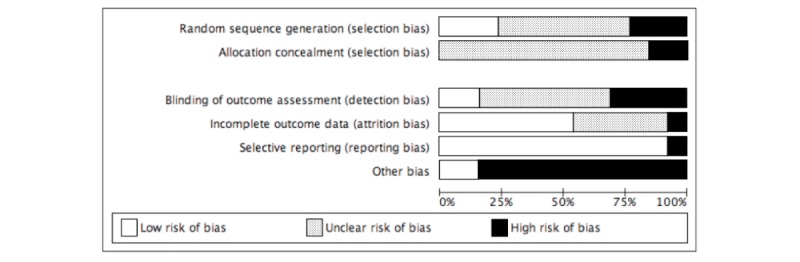
Risk of bias assessment.

Five studies referred to previously published study protocols [50,51,54,59,60]. These were used to obtain missing information needed for the risk of bias assessment. The judgment of each risk of bias item across studies can be found in Figure 2. Tables 4 and 5 show the support for judgment of each item and study.

Several studies were rated as unclear selection bias with regard to random sequence allocation [48,50,51,54-57]. Three were rated high risk [52,53,59], and 3 were rated low risk [49,58,60]. Most studies also tended to be of unclear risk of selection bias with regard to allocation concealment [48-51,53-58,60]. Two studies were rated as high risk for this item [52,59]. A total of 7 studies were ranked to be of unclear risk of detection bias [20,21,23-26,30], with 4 judged as high-risk [50,54,55,59] and 2 as low-risk [56,58]. With regards to attrition bias, 7 studies were judged to be of low risk [50,51,53-56,59], whereas 5 were ranked as unclear [49,52,57,58,60] and one as high-risk [48]. Twelve studies were of low risk of reporting bias [48-57,59,60]. Only one study was classed as high risk of bias for this item [58]. Ten studies were ranked as high risk of response and recall bias [49-54,56-58,60]. Risk of compliance bias was evident in 3 studies [48,49,53]. Another study was judged to be of high risk of analytical bias [58]. Two studies appeared free of other sources of bias [55,59].

### Synthesis of Results

PA and SB assessed in hours per week or hours per day were converted into min per week and min per day [52,57]. For the following, intervention group refers to those involving SMS text messages. An overview of the findings including PA and SB outcomes and outcome measures can be found in Table 6. Table 7 shows theoretical frameworks used and effectiveness of intervention groups in each study.

**Table 4:** Support for judgment of risk of bias per item and study. Random sequence generation, allocation concealment, and blinding of outcome assessment.

Author, year	Random sequence generation	Allocation concealment	Blinding of outcome assessment
Brannon et al, 2017 [48]	Unclear; Not enough information	Unclear; Not enough information	Unclear; Not enough information
Chen et al, 2017 [49]	Low; Randomization using computer program	Unclear; Not enough information	Unclear; Not enough information
Dewar et al, 2013 [50]	Unclear; Not enough information	Unclear; Not enough information	High; At baseline only. Outcomes likely to be influenced by lack of blinding
Dewar et al, 2014 [51]	Unclear; Not enough information	Unclear; Not enough information	Unclear; Not enough information
Ermetici et al, 2016 [52]	High; No randomization	High; No randomization	Unclear; Not enough information
Lana et al, 2014 [60]	Low; Randomization using computer program	Unclear; Not enough information	Unclear; Not enough information
Lau et al, 2012 [53]	High; No randomization	Unclear; Not enough information	Unclear; Not enough information
Lubans et al, 2012 [54]	Unclear; Not enough information	Unclear; Not enough information	High; At baseline only. Outcomes likely to be influenced by lack of blinding
Mendoza et al, 2017 [55]	Unclear; Not enough information	Unclear; Not enough information	High; Unblinded RCT^a^
Newton et al, 2009 [56]	Unclear; Not enough information	Unclear; Not enough information	Low; Assessors blinded at follow-up
Patrick et al, 2013 [57]	Unclear; Not enough information	Unclear; Not enough information	Unclear; Not enough information
Sirriyeh et al, 2010 [58]	Low; Randomization using random number generator	Unclear; Not enough information	Low; Assessors blinded at follow-up
Straker et al, 2014 [59]	High; Within-subject waitlist study design	High; Within-subject waitlist study design	High; Outcomes likely to be influenced by lack of blinding

^a^RCT: randomized controlled trial.

### Physical Activity

Included studies assessed accelerometer counts [50,54], light PA [59], moderate or vigorous PA [48,50-59], step count [56], or the number of days when a minimum of 60 min of PA was achieved [49]. Nine studies assessed MVPA [48,50,52-58]. Three studies resulted in a decrease between baseline and longest follow-up for the intervention group [50,54,56,57]. One study, however, found an increase in MVPA between 6- and 12-month assessment [57]. In another study, MVPA of normal weight participants increased between baseline and 2-school-year follow-up for the intervention group, however, decreased for the control. For overweight or obese participants, MVPA increased in both groups [52]. Four interventions resulted in increases in MVPA for all intervention and control groups between baseline and follow-up [53,55,56,58]. Two studies assessing MVPA used different types of SMS [48,58]. TMs sent by parents were effective in increasing MVPA for 70% of participants, SMS sent by a peer for 50%, and those sent from a behavioral health specialist for 90% of participants. Overall, the intervention resulted in higher levels of PA than during the control condition [48]. Another study employed neutral, affective, instrumental, or a combination of affective and instrumental SMS [58]. Across all participants, MVPA increased during the 2-week intervention with affective SMS resulting in the highest levels of PA undertaken [58]. In 2 studies, MPA and vigorous PA (VPA) were assessed [51,59]. Total, during school, after school, and weekday MPA and VPA decreased from baseline to 12-week follow-up for both intervention and control group [51]. The other study showed increases in MPA and VPA between baseline and 12 months [59].

**Table 5:** Support for judgment of risk of bias per item and study. Incomplete outcome data, reporting bias, and other bias.

Author, year	Incomplete outcome data	Reporting bias	Other bias
Brannon et al, 2017 [48]	High; High amount of missing data	Low; All outcomes reported	High; Compliance bias (use of incentives)
Chen et al, 2017 [49]	Unclear; Insufficient reporting of reasons for missing data	Low; All outcomes reported	High; Response bias (use of self-report), compliance bias (use of rewards)
Dewar et al, 2013 [50]	Low; Missing outcome data balanced and similar reasons across groups	Low; All outcomes reported	High; Response bias (use of self-report)
Dewar et al, 2014 [51]	Low; Missing outcome data balanced and similar reasons across groups	Low; All outcomes reported	High; Response bias (use of self-report)
Ermetici et al, 2016 [52]	Unclear; Insufficient reporting of reasons for missing data	Low; All outcomes reported	High; Response bias (use of self-report)
Lana et al, 2014 [60]	Unclear; Insufficient reporting of attrition, exclusions, and reasons	Low; All outcomes reported	High; Response bias (use of self-report)
Lau et al, 2012 [53]	Low; Missing outcome data balanced and similar reasons across groups	Low; All outcomes reported	High; Response bias (use of self-report), compliance bias (use of incentives)
Lubans et al, 2012 [54]	Low; Missing outcome data balanced and similar reasons across groups	Low; All outcomes reported	High; Response bias (use of self-report)
Mendoza et al, 2017 [55]	Low; Missing outcome data balanced and similar reasons across groups	Low; All outcomes reported	Low; Appears free of other sources of bias
Newton et al, 2009 [56]	Low; Missing outcome data balanced and similar reasons across groups	Low; All outcomes reported	High; Response bias (use of self-report)
Patrick et al, 2013 [57]	Unclear; Insufficient reporting of reasons for exclusions and dropouts	Low; All outcomes reported	High; Response bias (use of self-report)
Sirriyeh et al, 2010 [58]	Unclear; Insufficient reporting of reasons for exclusions and dropouts	High; Missing mean and SD of MET^a^ min at time point 1	High; Response bias (use of self-report), analytical bias (removal of outliers)
Straker et al, 2014 [59]	Low; Missing outcome data balanced and similar reasons across groups	Low; All outcomes reported	Low; Appears free of other sources of bias

^a^MET: metabolic equivalent.

For the intervention group, one study found an increase in PA levels between baseline and 3 months and between baseline and 6 months. PA levels decreased in the control condition [49]. Assessments of accelerometer counts, light PA, and daily step count showed decreases between baseline and follow-up [50,54,56,59].

### Sedentary Behavior

Studies assessed screen time [49,50,52,54], total SB [48,51,55,57,59], and whether participants performed less than 360 min of PA per week [60]. Three interventions found a decrease in screen time between baseline and longest follow-up [49,50,52]. One study found an increase in subjectively measured screen time on weekdays, however, a decrease on weekends [54]. In one intervention [51], subjective SB decreased in the intervention group and increased in the control group between baseline and 12 months. However, objectively measured SB increased for both groups. In 2 studies [55,57], the intervention groups reduced their total SB between baseline and follow-up, whereas the usual care or control group showed an increase in SB. Another intervention found an increase in SB between baseline and 8 weeks, 3 months, 6 months, and 12 months [59]. One intervention resulted in an increase in insufficient PA in the intervention group between baseline and 9 months, although, both the control groups reduced their level of insufficient PA during the same period [60]. In another study, SB was the lowest when receiving SMS from a parent but was the highest when receiving them from a behavioral health specialist, followed by SMS from a peer [48].

**Table 6:** Overview of physical activity (PA) and sedentary behavior (SB) outcomes and outcome measures in intervention groups at longest follow-up.

Outcome category		Accelerometer	Pedometer	Questionnaire	Interview
**Physical activity outcomes**					
	Accelerometer counts/min	Decrease [50]	—	—	—
	Light PA min/day	Decrease [59]	—	—	—
	MVPA^a^ %	Decrease [50]	—	—	—
	MVPA min/week		—	Increase [52,56]	Decrease [57]
	MVPA min/day	Increase [48,55]; decrease [54]	—	—	—
	MPA^b^ %	Decrease [51]	—	—	—
	MPA min/day	Increase [59]	—	—	—
	VPA^c^ %	Decrease [51]	—	—	—
	VPA min/day	Increase [59]	—	—	—
	MVPA score	—	—	Increase^d^ [53]	—
	4-day step count	—	Decrease [56]		—
	MVPA MET^e^ min/week	—	—	Increase [58]	—
	PA days/week	—	—	Increase^f^ [49]	—
**Sedentary behavior outcomes**					
	Screen time min/day	—	—	Decrease [50,52]; increase and decrease [54]	—
	Television/computer hours/day	—	—	Decrease^f^ [49]	—
	Total SB	Increase [51,59]; increase and decrease [48]; decrease [55]	—	Decrease^d^ [51]; decrease [57]	—
	PA less than 360 min/week	—	—	Increase [60]	—

^a^MVPA: moderate to vigorous physical activity.

^b^MPA: moderate physical activity.

^c^VPA: vigorous physical activity.

^d^Statistically significant (*P*<.05) between baseline and longest follow-up.

^e^MET: metabolic equivalent.

^f^Statistically significant (*P*≤.01) between baseline and longest follow-up.

**Table 7:** Theoretical framework and intervention effectiveness for intervention group at longest follow-up for individual studies.

Outcome category		TTM^a^	TPB^b^		SCT^c^		SDT^d^		CCT^e^	N/A^f^	
**Physical activity**											
	Brannon et al, 2017 [48]	—		—		—		—	P^g^		—
	Chen et al, 2017 [49]	—		—		P^h^		—	—		—
	Dewar et al, 2013 [50]	—		—		N^i^		—	—		—
	Dewar et al, 2014 [51]	—		—		N		—	—		—
	Ermetici et al, 2016 [52]	—		—		—		—	—		P
	Lau et al, 2012 [53]	P^h^		—		—		—	—		—
	Lubans et al, 2012 [54]	—		—		N		—	—		—
	Mendoza et al, 2017 [55]	—		—		—		P	—		—
	Newton et al, 2009 [56]	—		—		—		—	—		N
	Patrick et al, 2013 [57]	N		—		—		—	—		—
	Sirriyeh et al, 2010 [58]	—		P		—		—	—		—
	Straker et al, 2014 [59]	—		—		—		P	—		—
**Sedentary behavior**		—	—		—		—		—	—	
	Brannon et al, 2017 [48]	—		—		—		—	N, P		—
	Chen et al, 2017 [49]	—		—		P^h^		—	—		—
	Dewar et al, 2013 [50]	—		—		P		—	—		—
	Dewar et al, 2014 [51]	—		—		N^j^, P^j^		—	—		—
	Ermetici et al, 2016 [52]	—		—		—		—	—		P
	Lana et al, 2014 [60]	N		—		—		—	—		—
	Lubans et al, 2012 [54]	—		—		P		—	—		—
	Mendoza et al, 2017 [55]	—		—		—		P	—		—
	Patrick et al, 2013 [57]	P		—		—		—	—		—
	Straker et al, 2014 [59]	—		—		—		N	—		—

^a^TTM: transtheoretical model.

^b^TPB: theory of planned behavior.

^c^SCT: social cognitive theory.

^d^SDT: self-determination theory.

^e^CCT: cybernetic control theory.

^f^N/A: no theory framework.

^g^P: positive effect (PA increase, SB decrease).

^h^Statistically significant (*P*≤.01) between baseline and longest follow-up.

^i^N: negative effect (PA decrease, SB increase).

^j^Statistically significant (*P*<.05) between baseline and longest follow-up.

## Discussion

### Summary of Evidence

This review found promising evidence regarding the effectiveness of interventions using SMS to improve PA and SBs. Out of 5 studies assessing MVPA via self-report, 4 found an increase in PA [52,53,56,58] whereas for objectively assessed MVPA, 2 interventions showed an increase [48,55] and one a decrease [50,54]. Four studies resulted in a decrease for objectively assessed accelerometer counts, light PA, MPA, VPA, and step count [50,51,56,59]. One intervention showed an increase in objectively measured MPA and VPA [59]. Five studies assessing screen time and total SB using questionnaires demonstrated improvements [49-52,57], whereas objectively measured total SB increased in 3 [48,51,59] and decreased in 2 studies [48,55]. Of 10 interventions involving PA assessment, 8 resulted in an improvement of at least one PA outcome and of 8 assessing SB outcomes, 5 showed improvements.

Most interventions included in this review focused on increasing PA, whereas elements targeting SB were scarce. Evidence suggests that distinct assessment and approaches are required to improve PA and SB [61,62]. Previous meta-analyses have shown greater SB improvements in interventions solely targeting SB compared with PA interventions or those combining PA and SB [63,64]. To maximize intervention effectiveness, future studies should consider using distinct approaches to improve SB and PA.

The evidence presented in this review noted a variety of different outcome measures, which led to conflicting findings. For both PA and SB, more studies showed improvements when using subjective measures compared with objective measures. This is in line with previous findings showing subjective measures demonstrate greater enhancements than objective measures [65]. As self-report measures demonstrate low to moderate validity for the assessment of PA in children and adolescents, it appears that to assess effectiveness, objective measures such as accelerometers are preferred for both PA and SB [66]. For the assessment of the nature and mode of activity being undertaken, subjective measures should be used [61,66]. Further, a variety of protocols for the assessment and evaluation of participant data has been used. It has been shown that the choice of data reduction protocol when analyzing accelerometer data has a significant effect on the classification of SB and PA time in children [67]. There is a continued need for the standardization of methods when using objective measures to assess PA and SB [61], and future studies should consider following current recommendations on the assessment of both PA and SB to enhance the comparability of findings between studies and allow more distinct and unbiased conclusions to be drawn.

Identified studies also used a variety of theoretical frameworks with the more frequent use of the TTM and SCT, consistent with the findings of others [29]. Interventions informed by SDT, TPB, or CCT showed improvements in PA, whereas interventions informed by the TTM, SCT, and CCT revealed mixed results for PA and SB. Interventions employing SCT showed more positive results for SB than for PA. Nonetheless, the lack of information provided on how theory was applied within the intervention precludes our ability to confirm these assumptions with certainty. These findings are in line with those of a recent meta-analysis [44] that stated it was unclear how specific theoretical frameworks are applied or how they are linked to intervention effectiveness. Thus, our findings do not allow for a judgment on whether the ineffectiveness of some interventions included in this review is due to a lack of appropriate theory derivation and application. Furthermore, conclusions with regard to how theory relates to intervention effectiveness need to be drawn with caution, and more evidence is needed to warrant the use of specific theories when targeting PA and SB in SMS text messaging–based interventions for youth.

Evidence has shown the increased effectiveness of PA and SB interventions that include the BCTs of goal-setting, self-monitoring, and feedback [68]. In this review, 7 studies included goal-setting and monitoring, with 5 showing an increase in PA [48,49,53,55,59]. Two studies additionally included feedback and achieved improvements in PA [48,53]. Four studies that included self-monitoring and goal-setting found an improvement in SB [48,49,55,57]. These results are promising and indicate increased intervention effectiveness when including these BCTs in SMS-based interventions targeting PA and SB.

Previous reviews have shown weaknesses in the design of mHealth interventions [28,29,36,44]. Our findings were in agreement with those reviews and suggest that SMS-based interventions involving adolescents are weak in design and at a high risk of bias. The reasons for high risk of bias were attributed to the use of self-report measures (response bias), a lack of appropriate randomization method (selection bias), and a lack of blinding (detection bias).

We were also unable to infer the independent effect of SMS due to the lack of appropriate control groups. Only 4 studies employed designs that allowed for the effect of SMS text messaging alone to be assessed [48,57,58,60]. Two studies showed a positive effect of SMS on PA [48,58] and 2 on SB [48,57]. However, most studies included a variety of additional intervention components alongside SMS in the intervention and control groups. Definite conclusions with regard to the effectiveness of individual intervention designs, settings, or contents can therefore not be drawn from this review. Future research should employ study designs that allow the examination of the independent effect of SMS on PA and SB to strengthen the evidence base regarding the effectiveness of using SMS alone. Additionally, there is a need for studies exploring which specific SMS text messaging components such as content or frequency of delivery are most effective.

There is also a continued demand for studies to explore long-term intervention effects on PA and SB [24,28,32,35,37,43]. Only 4 interventions lasted for 12 months or longer [50-52,54,57,59]. Two studies assessed PA and SB after 24 months [50,52], with only one showing improvements in PA [52] but both showing decreases in SB [50,52]. It has been shown that SMS may be an effective tool to enhance participants’ interest in the long term as well as to improve adherence [31,36]. Therefore, more studies should explore the effectiveness of interventions in achieving sustained behavior change.

This review shows a high heterogeneity of study designs, intervention components, outcomes, and outcome measures. Possible conclusions regarding effective intervention designs and contents are limited and should be drawn with caution. This review provides some currently limited evidence that the following approaches may result in increased effectiveness of SMS-based interventions for PA and SB in youth:

1. Specific focus on the desired behavior
2. Include self-monitoring, goal setting, and feedback components
3. Send 3 or more SMS per week for PA.

Furthermore, future research should incorporate the following methodological elements:

1. Use of objective outcome measures
2. Include long-term follow-up
3. Designs that allow assessing the independent effect of SMS.

### Limitations

The authors were unable to conduct a quantitative data analysis due to high heterogeneity of included studies and a small pool of suitable data consisting of highly heterogeneous interventions and outcome measures. This review included all studies incorporating SMS text messaging as part of their intervention, which resulted in a variety of intervention designs and contents. Consequently, we were unable to draw conclusions with regard to specific intervention elements positively influencing PA and SB. To the best of our knowledge, this review provides the first account of interventions using SMS targeting PA and SB in adolescents. It provides researchers and practitioners with a database of potentially effective components crucial to the development of successful behavior change interventions.

Existing reviews have employed methods to identify and code theory-based elements such as behavior change techniques of included studies [26,28,65]. This review has refrained from following this process for studies not specifying theory base. However, the authors of those studies were contacted and a lack of theoretical foundation was confirmed. Despite the possibility that these interventions were unintentionally and unknowingly based on theory, there was no overt application of theory to study design. Therefore, it is judged to have limited contribution to intervention effectiveness.

This review does provide a detailed account of the use of theory in SMS-based interventions involving adolescents that, to the best of our knowledge, is novel and crucial for understanding current trends in intervention design and content. Moreover, a rigorous methodology was used for acquiring suitable studies, as well as during the data extraction process. This included hand-searching bibliographies, contacting authors of eligible studies, following recognized guidelines during data extraction, and pilot-testing data extraction items. Existing reviews on technology-based interventions targeting health behavior change have failed to include one or more of these components [24-31,33,35,37,43,44].

### Conclusions

This review shows a high level of heterogeneity within SMS-based interventions targeting adolescent PA and SB. The evidence base consists of studies using different objective and self-report outcome measures that employ a variety of protocols, which impairs the ability to synthesize study content and results. Additionally, assessment of the risk of bias showed some limitations in the study and intervention design. Results of the individual as well as across studies should therefore be analyzed with caution. Future research should employ more rigorous research designs, more structured and coherent intervention components, as well as more appropriate and valid outcome measures. Overall, the findings of this study indicate that multicomponent interventions incorporating SMS can be effective in improving PA and SB in adolescents; however, more evidence is needed to further warrant SMS interventions to improve PA and SB.

## Abbreviations

BCT: behavior change technique
CCT: cybernetic control theory
MET: metabolic equivalent
mHealth: mobile health
MPA: moderate physical activity
MVPA: moderate to vigorous physical activity
PA: physical activity
SB: sedentary behavior
SCT: social cognitive theory
SDT: self-determination theory
SMS: short message service
SOC: stage of motivational readiness for change
TPB: theory of planned behavior
TTM: transtheoretical model
VPA: vigorous physical activity
